# MVA.85A Boosting of BCG and an Attenuated, *phoP* Deficient *M. tuberculosis* Vaccine Both Show Protective Efficacy Against Tuberculosis in Rhesus Macaques

**DOI:** 10.1371/journal.pone.0005264

**Published:** 2009-04-15

**Authors:** Frank A. W. Verreck, Richard A. W. Vervenne, Ivanela Kondova, Klaas W. van Kralingen, Edmond J. Remarque, Gerco Braskamp, Nicole M. van der Werff, Ariena Kersbergen, Tom H. M. Ottenhoff, Peter J. Heidt, Sarah C. Gilbert, Brigitte Gicquel, Adrian V. S. Hill, Carlos Martin, Helen McShane, Alan W. Thomas

**Affiliations:** 1 Department of Parasitology, Biomedical Primate Research Centre, Rijswijk, the Netherlands; 2 Animal Science Department, Biomedical Primate Research Centre, Rijswijk, the Netherlands; 3 Department of Pulmonology, Leiden University Medical Centre, Leiden, the Netherlands; 4 Department of Infectious Diseases, Leiden University Medical Centre, Leiden, the Netherlands; 5 The Jenner Institute, University of Oxford, Oxford, United Kingdom; 6 Institut Pasteur, Paris, France; 7 Centro de Investigación Biomédica en Red de Enfermedades Respiratorias (CIBERES), Faculty of Medicine, University of Zaragoza, Zaragoza, Spain; University of Hyderabad, India

## Abstract

**Background:**

Continuous high global tuberculosis (TB) mortality rates and variable vaccine efficacy of *Mycobacterium bovis* Bacille Calmette-Guérin (BCG) motivate the search for better vaccine regimes. Relevant models are required to downselect the most promising vaccines entering clinical efficacy testing and to identify correlates of protection.

**Methods and Findings:**

Here, we evaluated immunogenicity and protection against *Mycobacterium tuberculosis* in rhesus monkeys with two novel strategies: BCG boosted by modified vaccinia virus Ankara expressing antigen 85A (MVA.85A), and attenuated *M. tuberculosis* with a disrupted *phoP* gene (SO2) as a single-dose vaccine. Both strategies were well tolerated, and immunogenic as evidenced by induction of specific IFNγ responses. Antigen 85A-specific IFNγ secretion was specifically increased by MVA.85A boosting. Importantly, both MVA.85A and SO2 treatment significantly reduced pathology and chest X-ray scores upon infectious challenge with *M. tuberculosis* Erdman strain. MVA.85A and SO2 treatment also showed reduced average lung bacterial counts (1.0 and 1.2 log respectively, compared with 0.4 log for BCG) and significant protective effect by reduction in C-reactive protein levels, body weight loss, and decrease of erythrocyte-associated hematologic parameters (MCV, MCH, Hb, Ht) as markers of inflammatory infection, all relative to non-vaccinated controls. Lymphocyte stimulation revealed Ag85A-induced IFNγ levels post-infection as the strongest immunocorrelate for protection (spearman's rho: −0.60).

**Conclusions:**

Both the BCG/MVA.85A prime-boost regime and the novel live attenuated, *phoP* deficient TB vaccine candidate SO2 showed significant protective efficacy by various parameters in rhesus macaques. Considering the phylogenetic relationship between macaque and man and the similarity in manifestations of TB disease, these data support further development of these primary and combination TB vaccine candidates.

## Introduction

TB is an ancient infectious disease still afflicting mankind by causing mortality of over a 1.5 million deaths per year [1]. Increasing incidence of multi- and extensively drug resistant *M. tuberculosis* strains and co-infection with HIV make the situation worse. After several decades BCG (Bacille Calmette-Guérin), that is derived as an attenuated strain from *Mycobacterium bovis*, still is the only available TB vaccine today. However, BCG displays variable efficacy and poorly protects from the lung disease becoming mostly manifest during adulthood [2]. New and better therapies, including cost effective vaccination, are required to effectively fight TB.

Now, vaccines for use in combination with BCG and live vaccines to replace BCG are both being sought [3]–[5]. Recently, live recombinant Modified Vaccinia virus Ankara (MVA) expressing mycobacterial antigen 85A (MVA.85A) has shown protection when administered following BCG vaccination in small vertebrate models [6]–[8]. It enhances vaccine and naturally primed responses in humans and is currently undergoing Phase 2 clinical evaluation [9]–[13]. An alternative approach is to rationally design novel live attenuated *M. tuberculosis*. Here we use a novel vaccine candidate, SO2, constructed by disrupting the transcription regulator gene *phoP* implicated in *M. tuberculosis* virulence [14]. SO2 was recently shown to induce protection beyond the level of BCG in mouse and guinea pig models [8], [15], [16].

Non-human primates as phylogenetically the closest relatives to man and naturally susceptible to *M. tuberculosis* infection comprise a valuable model for investigating aspects of TB disease and protection [17]–[20], and for preclinical TB vaccine testing [21]–[24]. They can be considered to hold strong predictive validity and expected to support vital downselection of the most promising vaccines entering clinical efficacy testing.

Here, we used rhesus macacques (*Macaca mulatta*) as non-human primate hosts in a fixed-endpoint protocol including BCG-vaccinated and non-vaccinated animals as control groups, to demonstrate immunogenicity and protective efficacy of the MVA.85A booster and SO2 live attenuated vaccine candidates against *M. tuberculosis* challenge. Ex vivo lymphocyte stimulation testing with specific antigens and recording IFNγ secretion as a key mediator of protective immunity was performed to correlate specific responses to protection. The phylogenetically related rhesus macaque model, including correlates of TB disease, is further defined and holds potential for future identification of better endpoints for clinical efficacy testing.

## Results

### Vaccination, infectious challenge and treatment group characteristics

**Figure 1:**
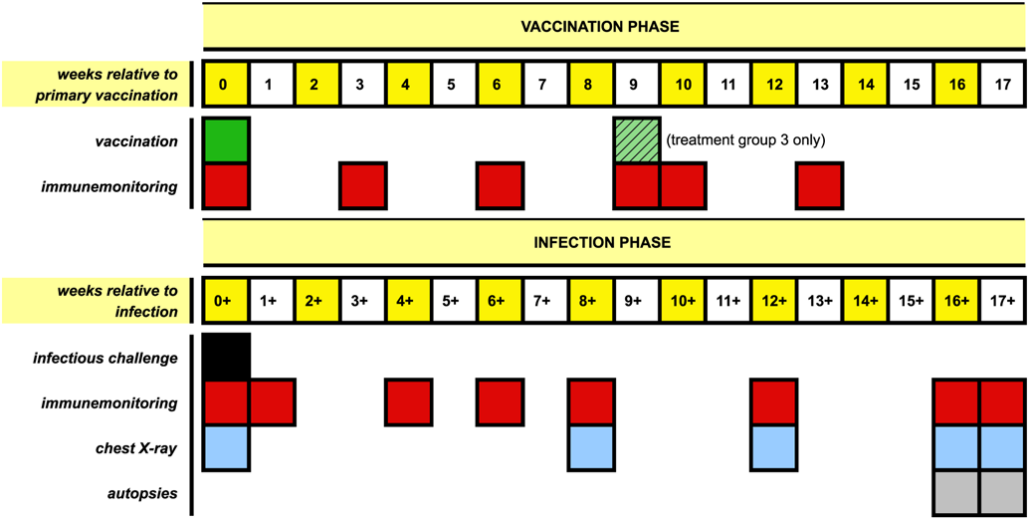
Experimental plan. A schematic diagram illustrating the timelines of vaccination (priming at week 0, boosting at week 9 for group 3 only), infectious challenge, immune monitoring, chest X-ray recording and fixed endpoint/autopsies by study protocol all relative to the 18 weeks immunisation phase or the 17/18 weeks challenge phase.

Vaccine treatments, dose and route of injection, and treatment group characteristics, number of animals (n), age and body weight, are summarised in Table 1. A schematic diagram representing the experiment is depicted in Figure 1. In brief, eighteen weeks after primary vaccination, animals were challenged in a single, randomised session by intratracheal instillation of 1000 colony forming units (CFU) of *M. tuberculosis* Erdman strain. The study plan accommodated a fixed endpoint with pathologic examination after euthanasia (by random sampling) at week 16/17 post-infection. Random stratification of animals over the four treatment groups warranted homogeneous distribution of body weight and age without significant differences between the groups at the start of the experiment (Table 1).

**Table 1:** Treatment groups, dosing, routing and group characteristics.

		Treatment				mean age	mean weight
	Treatment	Abbreviated	Dose	Route	n	years (95%CI§)	kg (95%CI§)
1	non-vaccinated	non-v	n.a.*	n.a.*	6	8.1 (7.2–8.9)	8.5 (7.2–9.8)
2	BCG vaccinated	BCG	5×10^5^ CFU	i.d.#	6	7.8 (7.1–8.4)	9.0 (6.8–11.1)
3	BCG primed and	B(CG)/MVA	5×10^5^ CFU	i.d.#	6	8.0 (6.9–9.1)	9.1 (7.3–10.9)
	MVA.85A boosted		5×10^8^ PFU	i.d.#			
4	SO2 vaccinated	SO2	5×10^5^ CFU	i.d.#	6	8.3 (7.9–8.9)	8.6 (6.9–10.2)

* n.a.: does not apply.

# i.d.: intradermally.

§ CI: confidence interval.

### Elevated antigen-specific IFNγ levels upon in vitro stimulation after vaccination showing effectivity of vaccination

**Figure 2:**
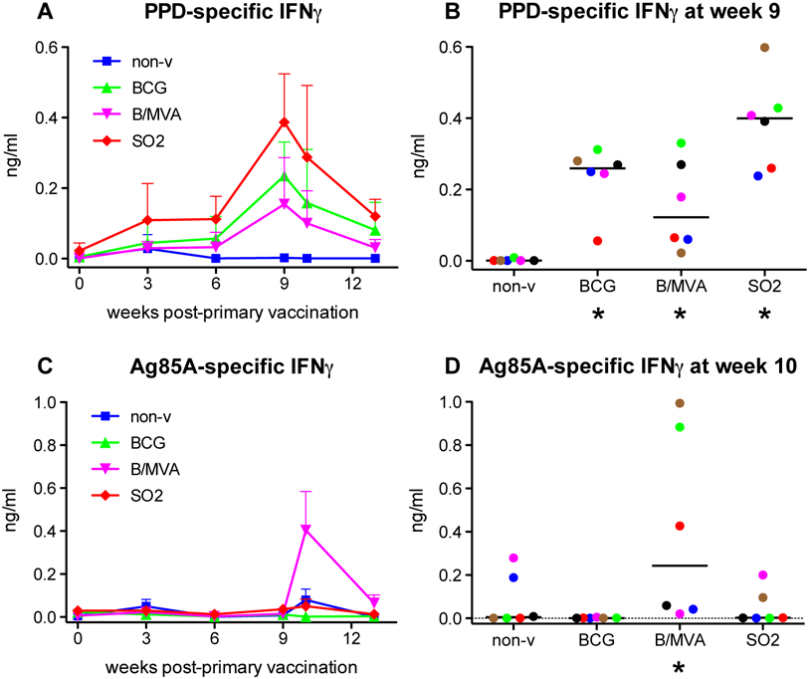
Mycobacterium-specific IFNγ secretion post-vaccination. Both the SO2 primary and the MVA.85A booster vaccine are immunogenic in rhesus macaques. Antigen-specific IFNγ secretion was measured after 3 days of in vitro stimulation of fresh peripheral blood lymphocytes along the vaccination phase. PPD-specific IFNγ secretion is depicted as group mean responses (+standard error) in time (A), and as individual responses with medians at the peak of the mean group response (B). Similarly, results upon stimulation with recombinant Ag85A are displayed as group means (+standard error) in time (C), and as individual responses (with medians) at peak (D), respectively. Colouring of group medians (panels A and C) is representing non-vaccinated controls in blue, BCG only in green, BCG/MVA.85A in magenta, and SO2 vaccination in red; group colouring is maintained throughout the paper for comparibility of data. Dot plots representing individual animals (panels B and D) are consistently coloured as follows: animals per treatment group were ranked from highest to lowest total gross pathology score (sum of lung, hilar LN and extra-thoracic scores) and then assigned the colours: blue, green, magenta, red, brown, black, respectively; individual colouring is maintained throughout for comparability. P-values are relative to non-vaccinated control treatment and represented under the x-axis by symbols as follows: ▪ for 0.1>p≥0.05, ★ for 0.05>p≥0.01, ★★ for 0.01>p≥0.001.

Following vaccination no clinically significant changes in behaviour, weight or parameters of hematology and clinical chemistry were evident in any group (data not shown). Mild swelling and redness occurred at the MVA injection site with self-limiting vesicle and crust formation not requiring medical attention and reminiscent of observations in human MVA vaccination [7]. All vaccinated groups, but not non-vaccinated controls, showed increased IFNγ secretion against *M. tuberculosis* purified protein derivative (PPD); responses were maximal at week 9 post-primary immunisation and highest in the SO2 group (Figure 2A and 2B). Antigen 85A-specific IFNγ secretion increased after booster immunisation with MVA.85A only (Figure 2C and 2D).

### TB-associated pathology and lesion scores upon autopsy revealed protective vaccine effects

All but three animals reached fixed protocol endpoint (week 16/17). Two non-vaccinated controls reached humane endpoint at week 12 post-infection due to sustained weight loss, and one BCG-vaccinee was withdrawn at week 14 post-infection after showing signs of distress. According to study plan these humanely culled animals, like those at fixed endpoint by protocol, underwent full pathologic examination at autopsy. All three showed active pulmonary TB and hilar lymph node (LN) involvement. The two non-vaccinated controls, but not the BCG vaccinee, presented with extra-pulmonary lesions (see below). Also, upon humane withdrawal defined clinical parameters (see below) were recorded to warrant a complete set of data for comprehensive analysis of 24 individuals at time of autopsy as an endpoint.

Upon pathologic examination most severe disease was generally observed in non-vaccinated controls. At autopsy all these control animals revealed lesions in their lungs and macroscopic pathologic findings were consistent with multi-focal to coalescing caseous and occasional non-necrotic, young nodules (tubercula). Histologically granulomas were apparent with or without necrotic centres, with neutrophils, and surrounded by epitheloid macrophages, scattered multi-nucleated giant cells, infiltrates of lymphocytes and plasma cells, and a peripheral rim of fibroblasts and collagen fibres. Beside specific tuberculous granulomatous lesions gross examination also revealed pulmonary changes including congestion in 6 out of 6 non-vaccinated controls, consolidation in 4 out of 6, pleural fibrinoid adhesions in 5 out of 6, oedema in 6 out of 6, and areas of emphysema in 2 out of 6 (Table 2). Hilar lymph nodes (LN) were grossly affected in all non-vaccinated controls. To evaluate possible dissemination of infection from the lung, spleen, liver, kidneys, heart, pancreas and gastrointestinal tract (GIT) were routinely inspected for gross appearance of lesions. In 4 out of 6 non-vaccinated controls any such extra-thoracic lesions (involving spleen, liver, kidney, heart and pancreatic lymph node) were apparent (Table 2).

**Table 2:** Summary of frequencies of TB-associated pathologic manifestations and total lesion scores per treatment group.

Treatment Group:	non-v	BCG	BCG/MVA	SO2
lung lesions (n/N)	6/6	5/6	3/6	5/6
congestion (n/N)	6/6	5/6	2/6	4/6
consolidation (n/N)	4/6	3/6	1/6	1/6
fibrinous pleuritis (n/N)	5/6	3/6	1/6	3/6
oedema (n/N)	6/6	5/6	1/6	3/6
emphysema (n/N)	2/6	3/6	1/6	1/6
hilar LN involvement (n/N)	6/6	5/6	4/6	6/6
extra-thoracic lesions (n/N)	4/6	1/6	1/6	2/6
sum of total PA lesion scores (arbitrary units)	142.0	63.5	39.0	61.0

Disease generally was less severe in BCG vaccinated control animals; one had no signs of gross lung lesions (Table 2). With exception of one, all BCG controls showed hilar lymph node (LN) involvement, and dissemination outside the thorax was observed in 1 of 6 BCG only animals and limited to the spleen.

BCG/MVA.85A vaccinated animals developed less severe disease and presented with fewer lesions than BCG only and naive controls: 3 out of 6 had no macroscopic pulmonary TB lesions; 2 of 6 showed no hilar LN involvement; only 1 of 6 displayed extra-thoracic dissemination to liver and spleen (Table 2). Microscopically, TB lesions were more fibrosed and confined, suggestive of healing (not shown).

Of the SO2 vaccinees 5 out of 6 showed macroscopic pulmonary lesions. However, disease was reduced compared to non-vaccinated controls, with less animals showing congestion, consolidation, fibrinous pleuritis or oedema (Table 2). Hilar LN were prominently involved in all SO2 animals; disseminated lesions apparent in only 2 of 6 involving spleen and liver (Table 2).

**Figure 3:**
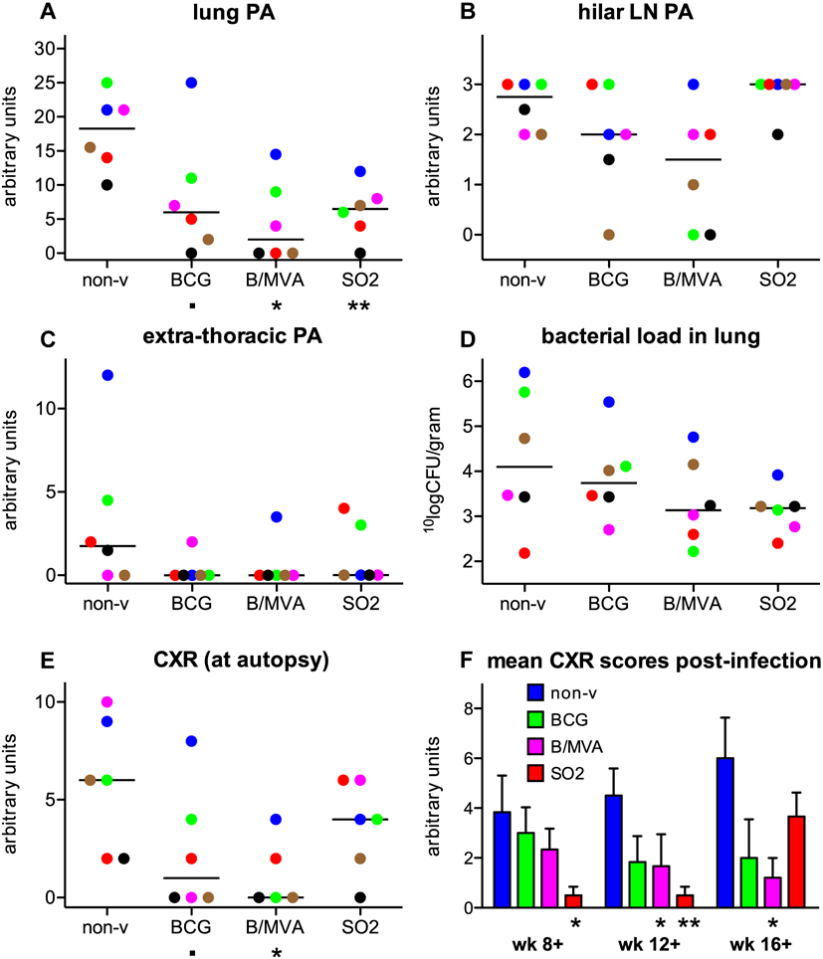
Evaluation of gross pathology, radiology and bacterial loads upon infectious challenge. Individual scores per treatment group and group median values are plotted for macroscopic lung pathology (PA) (A), hilar LN involvement (B), extra-thoracic PA (C), bacterial burden in the lung (D), and chest X-ray (CXR) scores at autopsy (E). Kinetics of TB disease are reflected by mean CXR scores (+standard error of the mean) per treatment group in time (F). BCG/MVA shows protection by significantly reduced lung lesion scores and CXR scores at autopsy compared to non-vaccinated controls, and by reduced hilar LN involvement and CFU in the lung (not significant). SO2 in comparison to non-vaccinated controls shows significant protection by reduced lung lesion scores and delayed kinetics of TB by CXR in time, and by reduced CFU in the lung (not significant). For legends of colouring and symbols see legend to Figure 2. For BCG/MVA treatment CXR at autopsy n = 5; one recording censored due to technical error.

**Figure 4:**
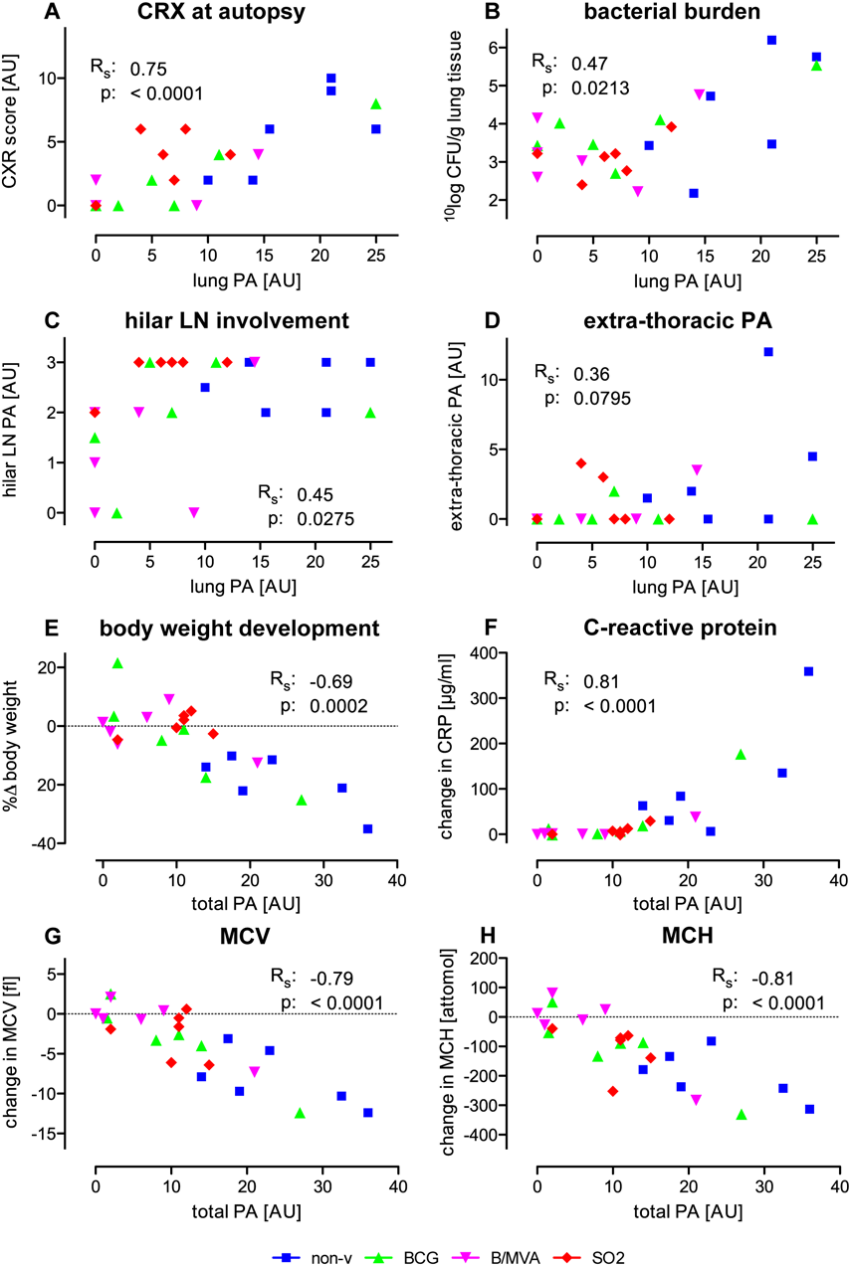
Correlations of disease along the infection phase. Thorax radiology, bacterial burden, and hilar LN pathology correlate significantly with lung PA. Loss of total body weight (wasting), and C-reactive protein (CRP) levels and decreasing mean corpuscular volume (MCV) and mean corpuscular hemoglobin (MCH) as measures of systemic inflammation, are highly significant correlates of disease in this high dose challenge model for TB vaccine evaluation. Parameters are plotted per individual (with colouring as indicated in the legend to Figure 2 on group colouring) against lung PA for CXR scores at autopsy (A), CFU counts from lung homogenates (B), hilar LN involvement (C), disseminated extra-thoracic lesions (D), and against total pathology (the sum of lung, hilar LN and extra-thoracic PA scores) for relative change in body weight (E), change in CRP (F), change in MCV (G) and in MCH (H). Spearman's rho (R_s_) as correlation factor and p-value are indicated. (AU for arbitrary units.)

Using a predefined arbitrary gross pathology (PA) scoring system for TB-associated lesions at autopsy, partial protection against challenge was demonstrated in all vaccine groups. Summed PA scores were 142, 63.5, 39 and 61 for non-vaccinated, BCG, BCG/MVA and SO2 groups, respectively (Table 2). As depicted in Figure 3A and 3C similar trends of reduced pathology after vaccination were observed for both lung PA and disseminated disease by extra-thoracic PA, respectively. The reduction in macroscopic lung PA was statistically signficant (p<0.05) for both BCG/MVA and SO2 treatment and approached significance (0.1>p>0.05) for BCG alone. Hilar LN pathology was particularly reduced after BCG/MVA vaccination (Figure 3B). Hilar LN involvement and extra-thoracic PA showed relatively weak correlation with gross lung PA at autopsy as calculated by non-parametric Spearman's rho (Rs) (Figure 4C and 4D, respectively).

### Chest X-rays post-infection indicated reduction of lung pathology or delay of disease kinetics after vaccination

As a means of non-invasive diagnostic assessment of TB-associated lung pathology we took conventional anterior-posterior chest X-rays (CXR) of the animals along the post-infection phase. A pre-defined arbitrary scoring algorithm was applied to obtain a semi-quantitative score of individual chest X-ray results at 8 and 12 weeks post-infection and immediately prior to autopsy. CXR at autopsy timepoints (Figure 3E), like gross lung PA scores (Figure 3A), revealed reduced median values for all vaccine groups in comparison to non-vaccinated controls. In comparison to non-vaccinated controls at endpoint (autopsy) BCG/MVA prime-boost treatment, but not SO2, revealed a median CXR score that was significantly lower, while CXR score after BCG control vaccination approached significance. The correlation between CXR results at autopsy and gross lung PA scores as calculated by non-parametric Spearman's rho (Rs) was strong (Rs = 0.75) and highly significant (p<0.0001) (Figure 4A).

Group averages of CXR scores in time are displayed in Figure 3F and reflect differential dynamics of lung disease between the treatment groups. While non-vaccinated controls showed progressive increase in CXR scores over the infection phase, both BCG and BCG/MVA showed decreasing CXR signals from week 8 post-infection onward. The SO2 vaccine group showed reduced CXR scores at weeks 8 and 12 post-infection, significantly lower than the non-vaccinated, but not BCG alone control group (Figure 3F). However, by the time of fixed endpoint of this study (week 16/17 post-infection by protocol) and unlike the reduction of PA and bacterial loads in the lung, this group displayed increased CXR scores similar to the level of those in non-vaccinated and BCG-primed animals at week 8 post-infection (Figure 3F). Together, CXR results for SO2 vaccination suggest a remarkable delay in the onset of TB lung disease after high-dose infectious challenge.

### Reduction of mycobacterial burden in the lung upon vaccination

A possible reduction in bacterial burden by counting colony forming units (CFU) from tissue is a measure for protective efficacy often assessed in experimental vaccine testing. To this end, we collected and minced the whole lung (after pathologic evaluation and histologic sampling) and further homogenised a random sample for CFU determination. The results are depicted as ^10^logCFU per gram of lung tissue in Figure 3D. In comparison to non-vaccinated controls BCG vaccination revealed an average reduction in bacterial load of 0.43 log, whereas BCG/MVA reduced CFU by 1.0 log and SO2 by 1.2 log. Bacterial load and lung PA correlated relatively weakly but significantly (Figure 4B).

### Protective vaccine effects by clinical measures of wasting and inflammation

**Figure 5:**
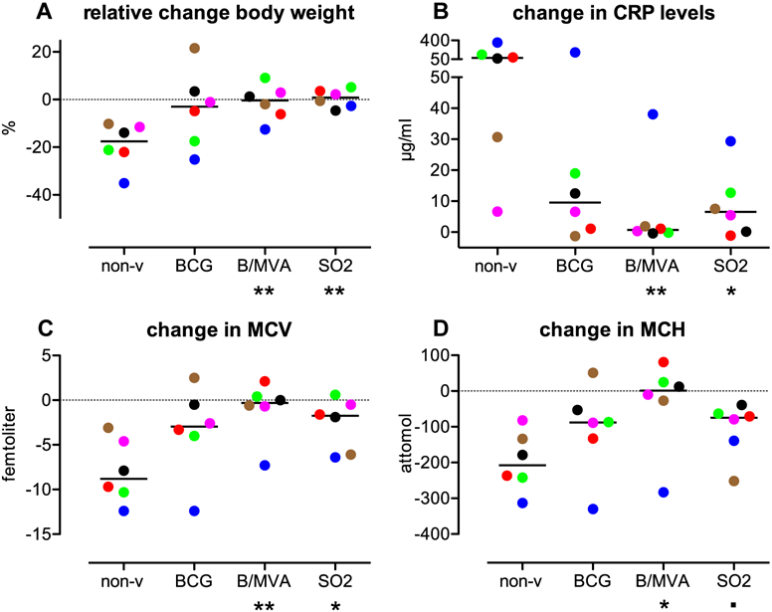
Evaluation of clinical measures along the infection phase. The BCG/MVA.85A regime and SO2 vaccination in comparison to non-vaccinated controls show significant protection from TB-associated wasting disease and systemic inflammation. Individual scores and group medians are plotted for relative change in body weight from start to end of the infection phase (A), change in C-reactive protein (CRP) levels (B), in mean corpuscular volume (MCV) (C), and in mean corpuscular hemoglobin (MCH) (D). For legends of colouring and symbols see legend to Figure 2.

Progressive tuberculosis in man is hallmarked by wasting disease and inflammation. Therefore, in this monkey experiment we measured changes in total body weight and several markers of systemic inflammation prior to and after infectious challenge. Wasting was reflected in the non-vaccinated controls by a (relative) loss of weight from pre-infection to the autopsy timepoint (Figure 5A). This weight loss was reversed by vaccination, and statistically significant improvement in comparison to non-vaccinated, but not to BCG controls was obtained for both BCG/MVA and SO2 (Figure 5A).

To assess systemic inflammation, levels of C-reactive protein (CRP) were measured in fresh serum samples along the infection phase and the absolute change in individual CRP levels from pre-infection to the autopsy timepoint is depicted in Figure 5B. While all animals typically displayed low CRP levels within normal ranges prior to infection (not shown), at autopsy the non-vaccinated control group showed systemic inflammation by a marked increase in CRP. Vaccination suppressed this inflammatory response and most significantly in the BCG/MVA group in which 5 out of 6 revealed no increase in CRP at all (Figure 5B).

Concordantly, erythrocyte-associated hematologic markers reflecting systemic inflammation such as decreasing values of mean corpuscular volume (MCV) and mean corpuscular hemoglobin (MCH) (Figure 5C and 5D, respectively) and related hematocrit (Ht) and hemoglobin (Hb) levels (not shown), corroborated the finding of systemic inflammation post-infection in the non-vaccinated control group. Again, vaccination suppressed this inflammatory response (Figure 5C and 5D). Like with CRP, live attenuated SO2 gave similar results as BCG for change in MCV and MCH. For SO2 vaccinees, but not for BCG, group medians of change in CRP and MCV were significantly different from those in non-vaccinated controls (Figure 5B and 5C), or approaching significance as for MCH (Figure 5D). MVA.85A vaccinated animals, except for one, showed no relevant decrease in MCV or MCH at all, and group medians were significantly different from the non-vaccinated, but not the BCG alone, control group.

These clinical measures of weight loss and change in CRP, MCV and MCH along the infection phase all displayed a strong and highly significant (non-parametric) correlation with total gross pathology (the sum of lung, hilar LN and extra-thoracic lesion scores) as depicted in Figure 4E, 4F, 4G and 4H, respectively.

### Antigen-specific IFNγ release post-infection and correlation of IFNγ secretory responses with pathology

**Figure 6:**
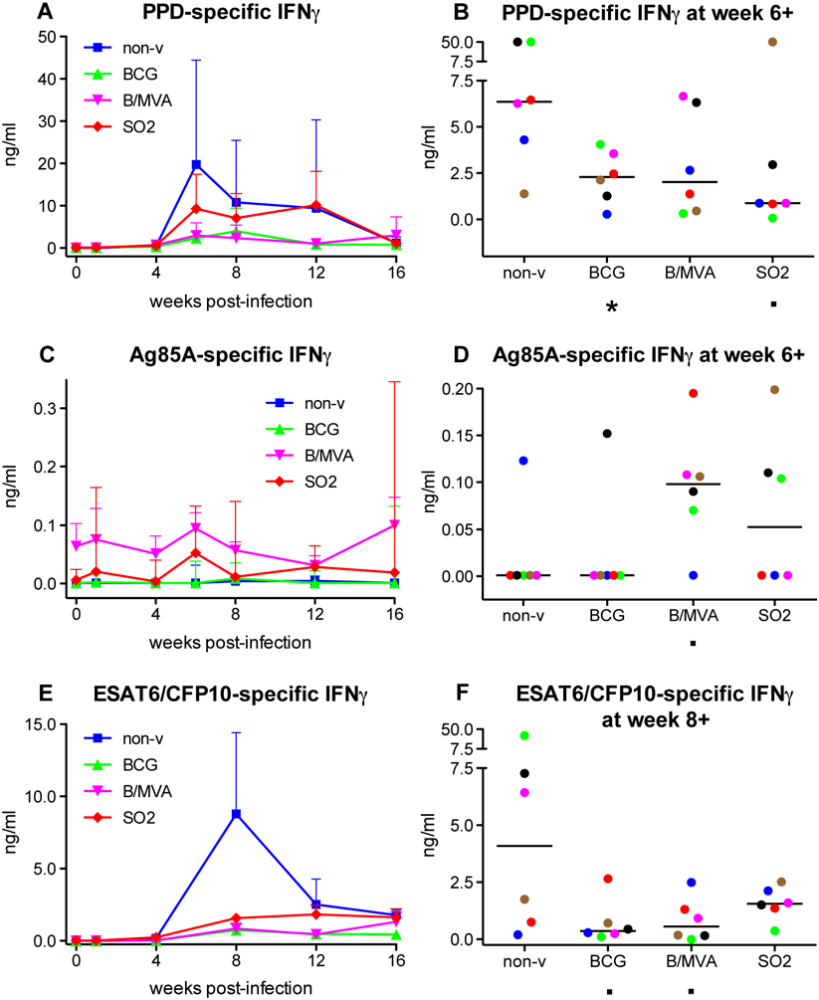
Mycobacterium-specific IFNγ secretion post-infection. Both PPD and ESAT6-CFP10 fusion protein, but not Ag85A, induce relatively high levels of IFNγ from in vitro stimulated fresh PBMC after infectious challenge with *M. tuberculosis*. Treatment group means (+standard errors) are plotted in time for PPD (A), Ag85A (C) and ESAT6/CFP10 fusion protein (E), and as individual responses (with group medians) at the peak of the mean response as indicated (B, D and F, respectively). For legends of colouring and symbols see legend to Figure 2.

Specific immune responses in the post-infection phase were monitored by measuring IFNγ secretion levels from fresh PBMC samples stimulated in vitro with PPD, recombinant Ag85A or ESAT6/CFP10 fusion protein. PPD- and ESAT6/CFP10 stimulation revealed highest IFNγ secretion in non-vaccinated controls in general at 6–8 weeks post-infection (Figure 6A and 6B, and 6E and 6F, respectively). In vaccine groups generally these responses were lower than in the non-vaccinated control group. In contrast, post-infection Ag85A specific IFNγ responses were relatively low (in the sub-nanogram range), and on average lower in non-vaccinated controls than in BCG/MVA and SO2 vaccine groups particularly (Fig 6C and 6D).

**Figure 7:**
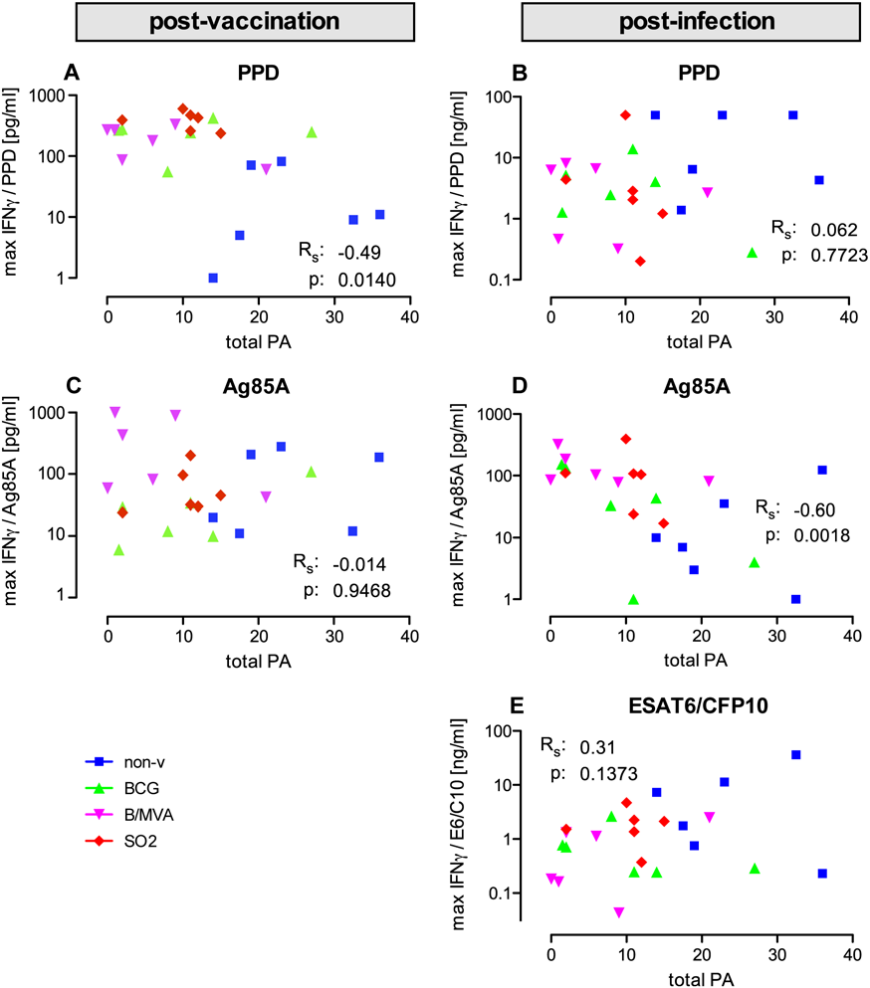
Immune correlations of disease. Maximal PPD-specific IFNγ levels post-vaccination and maximal Ag85A-specific IFNγ post-infection show significant inverse correlations with TB disease by total gross lesion PA scores. Maximal antigen-specific IFNγ response levels are plotted per individual against total pathology scores (with colouring as indicated in the legend to figure 2 on group colouring) and post-vaccination and post-infection for PPD (A and B, respectively) and Ag85A (C and D, respectively), and for ESAT6-CFP10 fusion protein post-infection only (E).

For both post-vaccination (pre-infection) and post-infection timepoints coefficients of correlation between individual maximal antigen specfic IFNγ levels and total PA lesion scores were calculated. Maximal PPD specific IFNγ levels post-vaccination (Figures 7A), but not post-infection (Figures 7B), showed a significant and inverse correlation with total PA (Rs = −0.49, p = 0.0140). Contrarily, maximal IFNγ release upon Ag85A stimulation post-infection (Figure 7D), but not post-vaccination (Figure 7C), correlated significantly with disease by total PA lesion score (Rs = −0.60, p = 0.0018). ESAT6/CFP10 specific maximal IFNγ secretion, which was monitored in the post-infection phase only, did not correlate significantly with total PA at autopsy (Figure 7E).

## Discussion

In the present study both a viral vaccine regime using recombinant MVA expressing Ag85A, subsequent to standard BCG primary vaccination, as well as a novel live attenuated *M. tuberculosis* strain SO2 used as a prime and only vaccine, showed induction of specific immunity and statistically significant protective effects from TB disease as observed in non-vaccinated control rhesus monkeys. Both experimental vaccines appeared safe and showed no relevant clinical adversity under the observational conditions of the experiment.

A relatively high dose infectious challenge with 1000 CFU of *M. tuberculosis* Erdman strain intratracheally triggered acute disease in the non-vaccinated control rhesus monkeys. This active TB disease was characterised by serious necrotic to coalescing lesions in the lungs and often involved other organs indicating disseminated disease. In accordance with lung PA, these non-vaccinated controls presented with gradually increasing lung lesion burden by longitudinal chest X-ray diagnosis. As in active human TB, these non-vaccinated rhesus macaques displayed wasting disease by loss of total body weight along the infection. Moreover, systemic inflammation as measured by increased C-reactive protein levels and by decreasing values for the erythrocyte-associated measures mean corpuscular volume and mean corpuscular hemoglobin (and the related hematocrit and hemoglobin concentration) was apparent.

Significant protective efficacy in comparison to non-vaccinated controls could be assigned by using non-parametric statistical evaluation (p<0.05) for both BCG/MVA and SO2 and by using 6 outbred animals per treatment group only. Protection was specifically reflected by significantly reduced gross lung lesion scores, CXR scores (but at varying timepoints for the different experimental regimes, see also below), wasting, and systemic inflammation as by CRP levels and the hematologic MCV/MCH values. Average CFU counts from the lungs were reduced by about 1 log compared to non-vaccinated controls, but not significant. Also, the BCG only control group, although not significant (p>0.05), showed protective effects by these parameters over non-vaccinated controls. However, under these conditions and using group sizes of 6, significant improvement beyond BCG could not be detected, neither with the MVA.85A vaccination nor the SO2 prime regime.

Generally, new TB vaccine strategies initially undergo evaluation to show protective efficacy in mouse and guinea pig models. One of those, the MVA.85A booster vaccine, to the best of our knowledge, is currently the most advanced in clinical safety trials [3], [11]. Here BCG/MVA demonstrates protective efficacy in non-human primates, supporting evaluation of the strategy in costly human efficacy trials. Improvement beyond BCG is suggested by further reduction of lung PA, hilar LN involvement, lung CFU, wasting and CRP levels, and more stable levels of MCV and MCH by study endpoint.

Data following SO2 vaccination of rhesus monkeys furthers proof of principle and extends the notion of this novel *phoP* deficient *M. tuberculosis* as a safe, immunogenic and protective vaccine candidate to the primate host. In comparison to BCG, this primary vaccine candidate showed similar protection by similar reduction of lung and extra-thoracic lesion scores, wasting disease and of systemic inflammation. While BCG on average reduced CFU in the lungs by 0.4 log, CFU were down by 1.2 log in the SO2 treated. Longitudinal CXR scores were reduced 8 and 12 weeks post-infection as compared to BCG and non-vaccinated controls. By the end of the study at week 16, however, CXR scores in SO2 were similar to those observed in the other groups at week 8. Together with the relatively strong hilar lymph node involvement in the SO2 vaccinees, this suggests that these animals only developed active TB by the endpoint by protocol to a level observed for BCG and non-vaccinated controls at week 8 already. While the data here indicate that SO2 shows a remarkable delay in the kinetics of TB in the face of a high dose challenge with 1000 CFU intratracheally and relative to BCG treated animals, further studies are needed to investigate whether disease would deteriorate or whether it would go ‘in remission’ like suggested by the CXR dynamics for the BCG vaccinated macaques in this study from week 8 post-infection onward.

Targeted disruption of *phoP* reduces ESAT6 secretion and expression of lipids implicated in virulence and immune modulation, retains immunodominant *M. tuberculosis* products missing from BCG, and provides more attenuation than BCG in SCID mice [15], [25]–[28]. Lung homogenates plated on selective medium did not reveal SO2 persistence in vaccinated animals, corroborating the SO2 safety profile (not shown). With regard to safety the next development step for SO2 requires the creation of an additional, independent mutation [29], [30]. Together, this *M. tuberculosis* derived live *phoP* gene knock-out SO2 vaccine candidate is the first of its kind, different from *M. bovis* derived BCG and rationally engineered to attenuation, that holds potential to protect the primate host. Generally, live mycobacterial vaccines may hold promise to replace and improve upon BCG as a single-dose primary vaccine in the future.

We have previously reported failure of BCG to protect against *M. tuberculosis* Erdman challenge in rhesus macaques, in contrast to findings here [18]. This apparent paradox is subject to ongoing investigation, this indicating animal origin, consequent to geographically restricted genetic/environmental factors (including pre-exposure), may significantly affect live vaccine outcomes. However, disease kinetics in rhesus macaques, as corroborated by chest X-ray results here, indicate that 8/9 weeks post-infection (the fixed endpoint for the former study) is too early to reveal protective effects in BCG vaccinated rhesus macaques. The delayed onset of lung pathology in SO2 vaccinated animals further suggests that endpoints beyond the 16 weeks post-infection used here are warranted.

Immune monitoring post-infection by using PBMC and in vitro stimulation with PPD or ESAT6-CFP10 fusion protein revealed reduced IFNγ release from vaccine groups as compared to non-vaccinated controls. Speculatively, this reduction of antigen-specific responsiveness suggests that vaccination may lead to reduced antigen-specific peripheral immune activity due to protection (i.e. reduced mycobacterial load and hence reduced immune stimulation), to regulatory responses suppressing IFNγ release, and/or to specific homing of antigen-specific lymphocytes to infected tissues post-infection in the vaccinated. Proof for any of these explanations and their possible relative contribution will require further in-depth investigations. In agreement with the concept of inducing a protective IFNγ positive cellular immune response by vaccination, maximal PPD-specific IFNγ levels post-vaccination showed an inverse and significant correlation with total gross pathology. Post-infection, however, this association by non-parametric evaluation by Spearman's rho could not be identified. In contrast to PPD or ESAT6/CFP10 induced responses post-infection, there is no sign of reduced Ag85A specific IFNγ in the vaccinated versus non-vaccinated groups. In addition, maximal IFNγ release levels upon Ag85A stimulation post-infection correlate well and inversely with disease outcome by gross pathology scoring. While the MVA.85A vaccine is specifically designed to enhance that response, apparently also SO2 as a primary vaccine allows for the detection of elevated Ag85A-specific IFNγ in the early post-infection phase. Thus, Ag85A-specific IFNγ may represent an interesting marker for future vaccine evaluations.

We demonstrate here the potential for BCG/MVA and SO2 vaccine strategies to protect phylogenetically related, naturally susceptible primates, providing support towards clinical assessment of these strategies. This study corroborates and extends the value of non-human primate TB models by further defining and correlating pathologic, radiologic, bacteriologic, clinical and hematologic read-outs, which improves vaccine selection prior to expensive and time-consuming clinical Phase IIb/III efficacy trials in high transmission areas. Elucidation of rhesus macaque and *M. tuberculosis* genomes [31], [32] provides opportunities to further identify mechanisms and (surrogate) markers of protection to accelerate TB vaccine development.

## Materials and Methods

### Ethics Statement

All animals in this study were captive bred for research purposes. BPRC housing and animal care procedures are in compliance with Dutch law on animal experiments, European directive 86/609/EEC, and with the “Standard for Humane Care and Use of Laboratory Animals by Foreign Institutions”, identification number A5539-01, provided by the Department of Health and Human Services of the US National Institutes of Health (NIH). The local independent ethical committee, constituted according to Dutch law on animal experiments, approved the study protocol prior to start of the experiment. Humane endpoints were pre-defined in this protocol and applied as a measure of reduction of discomfort.

### Animals

Healthy adult male rhesus monkeys (*Macaca mulatta*) were selected and randomly stratified on basis of age and body weight over 4 groups of 6 animals each. Monkeys were selected to be naive to prior mycobacterial exposure on the basis of a negative Mantoux skin reaction using *Old Tuberculin* (Statens Serum Institute (SSI), Copenhagen), a negative result in the Primagam® kit measuring *M.bovis/M.avium* PPD specific IFNγ release in whole blood cells (Biocor Animal Health, Omaha, Nebraska, USA), and clean chest X-rays lacking any signs of TB (see below).

### Animal handling

Animal handlings were performed after sedation (ketamine, 10 mg/kg). Heparinised blood for immune monitoring, EDTA blood for standard hematology, and serum for standard clinical chemistry were collected by venipuncture. Animal condition was monitored by daily observation and body weights recorded at all bleeding time points. Animals were challenged by intratracheal instillation of 1000 CFU of *M. tuberculosis* Erdman strain (kindly provided by P. Andersen c.s. from SSI, Copenhagen; cultured, aliquoted and stored by D. van Soolingen c.s. at RIVM, Bilthoven).

### Vaccines

*M.bovis* BCG (SSI, Copenhagen, Denmark; batch 10307D) was prepared as prescribed by the manufacturer. MVA.85A and SO2 vaccines are previously described [4], [13]. SO2 was diluted from stock solution in Sauton diluent prior to vaccination. All vaccines were applied by intradermal injection in the upper arm (booster vaccination in the opposite arm; timing and dosing as indicated).

### Autopsy and pathologic evaluation

For logistical reasons euthanasia by intravenous injection of sodium pentobarbital (Ethasol®, AST Farma, Oudewater) occurred at fixed time points on group sampling basis on 5 separate days (maximal interval of 8 days). At autopsy, gross pathology (PA) was scored while blinded for treatment according to an algorithm predefined by S. Reed, J.A. Flynn, and J.A.M. Langermans (unpublished). Lymph node involvement was scored as 1 (gross appearance of 1 lesion), 2 (several separate foci/necrotic areas) or 3 (extensive caseous necrosis). Five lung lobes were distincted and, like other organs, were scored as 1 (one gross lesion of <10 mm in diameter), 2 (two to five lesions <10 mm), 3 (more than five lesions of <10 mm or one lesion of >10 mm), 4 (more than one lesion of >10 mm), 5 (coalescing lesions); the total lung score was maximally 25. Histopathologic examination used formalin fixed and paraffin-embedded tissues according to regular procedures.

### Chest X-rays

CXR were obtained by exposure (76–80 Volt, 3 mAs) and using phosphor imaging plates, a CR2340 scanner, and Vision software (Vetray GmbH, Pfaffenhofen, Germany) for recording and reading. Readings were interpreted clinically and scored, while blinded to treatment, according to an algorithm adapted by K.v.K. from N. Mlika-Cabanne et al. [33]. Briefly, a semi-quantitative evaluation yields scores for each upper, middle and lower regions of the right and left lung on predominant infiltrates of 1 (small nodules), 2 (patchy appearance), 3 (consolidation). Besides, cavitation, pleural effusion and lymphadenopathy are scored (0, absent, 1 present) when it applies.

### Bacteriology

After collecting small representative samples for histopathology and cryopreservation, whole lungs were minced and a representative aliquot homogenised (PRO-200 homogenator; PRO Scientific, Oxford, CT). Diluted lung homogenates were plated on Middlebrook 7H10 medium plus OADC supplements and cycloheximid (100 µg/ml), (Tritium Microbiologie BV., Veldhoven, the Netherlands) to determine colony forming units (CFU) as a measure of bacterial load.

### Immune assays

Peripheral blood mononuclear cells (PBMC) were isolated freshly by density gradient centrifugation and immediately used in lymphocyte stimulation tests. Antigen-specific IFNγ secretion was assessed on duplicates in a non-human primate specific sandwich ELISA (U-CyTech, Utrecht) on pooled supernatants of triplicates of 2×10^5^ PBMC (per well) cultured for 3 days with and without antigen in RPMI 1640 culture medium supplemented with 10% (w/v) foetal calf serum and penicillin/streptomycin (Invitrogen; Leek, the Netherlands). Protein purified derivative of *M. tuberculosis* (RT49) (SSI; Copenhagen, Denmark), recombinant Ag85A and ESAT6/CFP10 fusion proteins were used at a final concentration of 10 µg/ml. Concanavalin A (Sigma, St. Louis) was used at 5 µg/ml as a positive control stimulus.

### Clinical assays

Standard parameters were determined for hematology (Sysmex XT 2000iV platform; Goffin Meyvis, Etten-Leur, the Netherlands) and clinical chemistry (Cobas Integra 400; Roche Diagnostics, Basel, Switzerland) on freshly obtained blood samples. C-reactive serum protein levels were determined using anti-CRP coated latex particles (Roche Diagnostics).

### Statistical analysis

Data were analysed by evaluating individual treatment groups by two-sided, non-parametric Mann-Whitney rank testing using Graph Pad Prism® software. Variance (R) between parameters and the p-values for R were calculated by non-parametric Spearman's rho also using Graph Pad Prism®. In general, results were considered significant for p-values smaller than 0.05, and for 0.1>p≥0.05 interpreted as approaching significance.

## References

[1] Global tuberculosis control: surveillance, planning, financing: WHO report 2008. WHO/HTM/TB/2008.393, at http://www.who.int/tb/publications/2008/en/index.html

[2] Fine PE (1995). Variation in protection by BCG: implications of and for heterologous immunity.. Lancet.

[3] Skeiky YAW, Sadoff JC (2006). Advances in tuberculosis vaccine strategies.. Nat Rev Microbiol.

[4] Baumann S, Nasser Eddine A, Kaufmann SH (2006). Progress in tuberculosis vaccine development.. Curr Opin Immunol.

[5] Hussey G, Hawkridge T, Hanekom W (2007). Childhood tuberculosis: old and new vaccines.. Paediatr Respir Rev.

[6] McShane H, Brookes R, Gilbert SC, Hill AVS (2001). Enhanced immunogenicity of CD4+ T-cell responses and protective efficacy of a DNA-modified vaccinia virus Ankara prime-boost vaccination regimen for murine tuberculosis.. Infect Immun.

[7] Goonetilleke NP, McShane H, Hannan CM, Anderson RJ, Brookes RH (2003). Enhanced immunogenicity and protective efficacy against *Mycobacterium tuberculosis* of bacille Calmette-Guerin vaccine using mucosal administration and boosting with a recombinant modified vaccinia virus Ankara.. J Immunol.

[8] Williams A, Hatch GJ, Clark SO, Gooch KE, Hatch KA (2005). Evaluation of vaccines in the EU TB Vaccine Cluster using a guinea pig aerosol infection model of tuberculosis.. Tuberculosis.

[9] McShane H, Pathan AA, Sander CR, Keating SM, Gilbert SC (2004). Recombinant modified vaccinia virus Ankara expressing antigen 85a boosts BCG-primed and naturally acquired antimycobacterial immunity in humans.. Nat Med.

[10] McShane H, Pathan AA, Sander CR, Goonetilleke NP, Fletcher HA (2005). Boosting BCG with MVA85a: the first candidate subunit vaccine for tuberculosis clinical trials.. Tuberculosis.

[11] Ibanga HB, Brookes RH, Hill PC, Owiafe PK, Fletcher HA (2006). Early clinical trials with a new tuberculosis vaccine, MVA85a, in tuberculosis-endemic countries: issues in study design.. Lancet Infect Dis.

[12] Pathan AA, Sander CR, Fletcher HA, Poulton I, Alder NC (2007). Boosting BCG with recombinant modified vaccinia ankara expressing antigen 85A: different boosting intervals and implications for efficacy trials.. PLoS ONE.

[13] Beveridge NE, Price DA, Casazza JP, Pathan AA, Sander CR (2007). Immunisation with BCG and recombinant MVA85A induces long-lasting, polyfunctional *Mycobacterium tuberculosis*-specific CD4+ memory T lymphocyte populations.. Eur J Immunol.

[14] Pérez E, Samper S, Bordas Y, Guilhot C, Gicquel B (2001). An essential role for phoP in *Mycobacterium tuberculosis* virulence.. Mol Microbiol.

[15] Martin C, Williams A, Hernandez-Pando R, Cardona PJ, Gormley E (2006). The live *Mycobacterium tuberculosis* phoP mutant strain is more attenuated than BCG and confers protective immunity against tuberculosis in mice and guinea pigs.. Vaccine.

[16] Aguilar D, Infante E, Martin C, Gormley E, Gicquel B (2007). Immunological responses and protective immunity against tuberculosis conferred by vaccination of Balb/C mice with the attenuated *Mycobacterium tuberculosis* (phoP) SO2 strain.. Clin Exp Immunol.

[17] Walsh GP, Tan EV, dela Cruz EC, Abalos RM, Villahermosa LG (1996). The Philippine cynomolgus monkey (*Macaca fascicularis*) provides new nonhuman primate model of tuberculosis that resembles human disease.. Nat Med.

[18] Langermans JA, Andersen P, van Soolingen D, Vervenne RAW, Frost PA (2001). Divergent effect of bacillus Calmette-Guerin (BCG) vaccination on *Mycobacterium tuberculosis* infection in highly related macaque species: implications for primate models in tuberculosis vaccine research.. Proc Natl Acad Sci USA.

[19] Flynn JL, Capuano SV, Croix D, Pawar S, Myers A (2003). Non-human primates: a model for tuberculosis research.. Tuberculosis.

[20] Capuano SV, Croix DA, Pawar S, Zinovik A, Myers A (2003). Experimental *Mycobacterium tuberculosis* infection of cynomolgus macaques closely resembles the varous manifestations of human M. tuberculosis infection.. Infect Immun.

[21] McMurray DN (2000). A nonhuman primate model for preclinical testing of new tuberculosis vaccines.. Clin Infect Dis.

[22] Langermans JA, Doherty TM, Vervenne RAW, van der Laan T, Lyashchenko K (2005). Protection of macaques against *Mycobacterium tuberculosis* infection by a subunit vaccine based on a fusion protein of antigen 85B and ESAT-6.. Vaccine.

[23] Kita Y, Tanaka T, Yoshida S, Ohara N, Kaneda Y (2005). Novel recombinant BCG and DNA-vaccination against tuberculosis in cynomolgus monkey model.. Vaccine.

[24] Sugawara I, Li Z, Sun L, Udagawa T, Taniyama T (2007). Recombinant BCG Tokyo (Ag85A) protects cynomolgus monkeys (*Macaca fascicularis*) infected with H37Rv *Mycobacterium tuberculosis*.. Tuberculosis.

[25] Friqui W, Bottai D, Majlessi L, Monot M, Josselin E (2008). Control of *M.tuberculosis* ESAT-6 secretion and specific T cell recognition by phoP.. PLoS Pathog.

[26] Gonzalo Asensio J, Maia C, Ferrer NL, Barilone N, Laval F (2006). The virulence-associated two-component PhoP-PhoR system controls the biosynthesis of polyketide-derived lipids in *Mycobacterium tuberculosis*.. J Biol Chem.

[27] Brosch R, Gordon SV, Garnier T, Eiglmeier K, Friqui W (2007). Genome plasticity of BCG and impact on vaccine efficacy.. Proc Natl Acad Sci USA.

[28] Gonzalo-Asensio J, Mostowy S, Harders-Westerveen J, Huygen K, Hernandez-Pando R (2008). PhoP: a missing piece in the intricate puzzle of *Mycobacterium tuberculosis* virulence.. PLoS ONE.

[29] Kamath AT, Fruth U, Brennan MJ, Dobbelaer R, Hubrechts P (2005). New live mycobacterial vaccines: the Geneva consensus on essential steps towards clinical development.. Vaccine.

[30] Asensio JA, Arbués A, Pérez E, Gicquel B, Martin C (2008). Live tuberculosis vaccines based on phoP mutants: a step towards clinical trials.. Expert Opin Biol Ther.

[31] Gibbs RA, Rogers J, Katze MG, Bumgarner R, Weinstock GM (2007). Evolutionary and biomedical insights from the rhesus macaque genome.. Science.

[32] Cole ST, Brosch R, Parkhill J, Garnier T, Churcher C (1998). Deciphering the biology of *Mycobacterium tuberculosis* from the complete genome sequence.. Nature.

[33] Mlika-Cabanne N, Brauner M, Mugusi F, Grenier P, Daley C (1995). Radographic abnormalities in tuberculosis and risk of coexisting human immunodeficiency virus infection. Methods and preliminary results from Bujumburra, Burundi.. Am J Respir Crit Care Med.

