# Correction: MVA.85A Boosting of BCG and an Attenuated, *phoP* Deficient *M. tuberculosis* Vaccine Both Show Protective Efficacy Against Tuberculosis in Rhesus Macaques

**DOI:** 10.1371/annotation/e599dafd-8208-4655-a792-21cb125f7f66

**Published:** 2011-02-26

**Authors:** Frank A. W. Verreck, Richard A. W. Vervenne, Ivanela Kondova, Klaas W. van Kralingen, Edmond J. Remarque, Gerco Braskamp, Nicole M. van der Werff, Ariena Kersbergen, Tom H. M. Ottenhoff, Peter J. Heidt, Sarah C. Gilbert, Brigitte Gicquel, Adrian V. S. Hill, Carlos Martin, Helen McShane, Alan W. Thomas

The following information was missing from the Funding section: "The study was supported by funding from the NIHR Oxford Biomedical Research Centre programme."

